# *LIPC* variants as genetic determinants of adiposity status, visceral adiposity indicators, and triglyceride-glucose (TyG) index-related parameters mediated by serum triglyceride levels

**DOI:** 10.1186/s13098-018-0383-9

**Published:** 2018-11-06

**Authors:** Ming-Sheng Teng, Semon Wu, Leay-Kiaw Er, Lung-An Hsu, Hsin-Hua Chou, Yu-Lin Ko

**Affiliations:** 1 Department of Research, Taipei Tzu Chi Hospital, Buddhist Tzu Chi Medical Foundation, New Taipei City, 23142 Taiwan; 2 Department of Life Science, Chinese Culture University, Taipei, 11114 Taiwan; 3 The Division of Endocrinology and Metabolism, Department of Internal Medicine, Taipei Tzu Chi Hospital, Buddhist Tzu Chi Medical Foundation, New Taipei City, 23142 Taiwan; 4 School of Medicine, Tzu Chi University, Hualien, 97071 Taiwan; 5 First Cardiovascular Division, Department of Internal Medicine, Chang Gung Memorial Hospital and Chang Gung University College of Medicine, Taoyuan, 33305 Taiwan; 6 Cardiovascular Center and Division of Cardiology, Department of Internal Medicine, Taipei Tzu Chi Hospital, Buddhist Tzu Chi Medical Foundation, New Taipei City, 23142 Taiwan

**Keywords:** Hepatic lipase, Single-nucleotide polymorphism, Triglyceride level, High-density lipoprotein cholesterol level, Adiposity status, Visceral adiposity indicators, TyG index-related parameters

## Abstract

**Background:**

Visceral adiposity indicators and the product of triglyceride and fasting plasma glucose (TyG) index-related parameters are effective surrogate markers for insulin resistance (IR) and are predictors of metabolic syndrome and diabetes mellitus. However, their genetic determinants have not been previously reported. Pleiotropic associations of *LIPC* variants have been observed in lipid profiles and atherosclerotic cardiovascular diseases. We aimed to investigate *LIPC* polymorphisms as the genetic determinants of adiposity status, visceral adiposity indicators and TyG index-related parameters.

**Methods:**

A total of 592 participants from Taiwan were genotyped for three *LIPC* single nucleotide polymorphisms (SNPs).

**Results:**

The *LIPC* SNPs rs2043085 and rs1532085 were significantly associated with body mass index (BMI), waist circumference (WC), lipid accumulation product, visceral adiposity index, and TyG index-related parameters [including the TyG index, TyG with adiposity status (TyG-BMI), and TyG-WC index], whereas the rs1800588 SNP was only significantly associated with the TyG index. The associations became nonsignificant after further adjustment for serum TG levels. No significant association was observed between any the studied *LIPC* SNPs and IR status.

**Conclusion:**

Our data revealed a pleiotropic association between the *LIPC* variants and visceral adiposity indicators and TyG index-related parameters, which are mediated by serum TG levels.

**Electronic supplementary material:**

The online version of this article (10.1186/s13098-018-0383-9) contains supplementary material, which is available to authorized users.

## Background

Insulin resistance (IR) is an insensitivity state of the peripheral tissue to the effects of insulin. IR and the consequence of compensatory hyperinsulinemia are fundamental pathogenic factors for various metabolic abnormalities [1] that contribute to the development of diabetes mellitus and cardiovascular disease [2, 3]. Hyperinsulinemic euglycemic pump remains the gold standard for measuring IR, whereas homeostatic model assessment for insulin resistance (HOMA-IR) index is more practically used for determining IR in clinical application. Recently, multiple predictors for IR have also been proposed. For example, the visceral adiposity index (VAI) is an indicator of adipose tissue dysfunction and a surrogate marker for IR, which is calculated on the basis of anthropometric body mass index (BMI), waist circumference (WC), and lipid traits [4, 5]. The lipid accumulation product (LAP), a mathematical model based on a combination of serum triglyceride (TG) levels and WC, is a sensitive marker for visceral obesity that has the potential to estimate IR [6, 7]. The product of TG and fasting plasma glucose (FPG) levels (the TyG index) and the TyG index-related parameters, including the TyG with adiposity status (TyG-BMI index) and the TyG-WC index (particularly the TyG-BMI), have been proposed as simple, efficient, and clinically useful surrogate markers for early identification of IR, which may further predict the occurrence of metabolic syndromes and diabetes mellitus [8–11].

Hepatic lipase (HL, encoded by *LIPC*) is a glycoprotein primarily synthesized and secreted by hepatocytes, and to a lesser extent, by macrophages and other tissues [12]. HL is a member of the triacylglycerol lipase family and is a key enzyme responsible for the hydrolysis of TGs and phospholipids in nearly all lipoprotein subclasses, resulting in the generation of small and dense particles [13]. HL also plays a role in the metabolism of high density lipoprotein (HDL) by converting large, TG-rich HDL_2_ into small, dense HDL_3_; moreover, it is a negative regulator of plasma HDL cholesterol (HDL-C) levels [12]. In humans, HL overexpression considerably reduces HDL-C levels because of the increased catabolic rate, whereas HL deficiency increases the levels of large HDL_2_ particles, enriches HDL with TG, and causes hyperalphalipoproteinemia because of slow apolipoprotein AI catabolism [13]. Andrés-Blasco et al. [14] also showed that HL-inactivation in mice fed with a high-fat, high cholesterol diet, exhibited augmented glucose levels for HL^−/−^ mice in feed state with similar serum insulin levels compared to wild type mice, suggesting glucose intolerance. In addition to its enzymatic functions, HL facilitates the uptake of chylomicron remnant-like particles by acting as a ligand for glycosaminoglycans on the surface of rat hepatocytes [13]. Overall, HL is crucial for reverse cholesterol transport, and it affects the lipoprotein and possibly glucose metabolism [14–16].

Recent studies have shown pleiotropic associations of *LIPC* single nucleotide polymorphisms (SNPs), which included lipid profiles, hepatic lipase activity, serum insulin levels, insulin sensitivity, markers for oxidative stress, metabolic syndrome and atherosclerotic cardiovascular diseases [17–26]. By contrast, controversial results have been reported on the association between *LIPC* SNPs and obesity [27–29]. The visceral adiposity indicators and TyG index-related parameters represent complex phenotypes with the combinations of adiposity status and metabolic traits. Therefore, in the present study, we aimed to investigate the association between *LIPC* SNPs and adiposity status, visceral adiposity indicators and TyG index-related parameters and IR in term of HOMA-IR in the Taiwanese population.

## Methods

### Participants

This study was approved by the Institutional Review Board of the Taipei Tzu Chi Hospital, Buddhist Tzu Chi Medical Foundation (IRB No. 02-XD39-090). After obtaining informed consent from the study participants, they were recruited consecutively during cardiovascular health examinations between October 2003 and September 2005 at Chang Gung Memorial Hospital. Exclusion criteria included a history of myocardial infarction, stroke, or transient ischemic attack; a history of cancer; and current renal or liver disease. Initially, 617 individuals were recruited. Participants who were younger than 18 years or who took medications for dyslipidemia and diabetes mellitus were further excluded. Finally, 592 Han Chinese participants (310 men with a mean age of 45.0 ± 9.6 years, and 282 women with a mean age of 46.7 ± 9.7 years) were enrolled for analysis. Current smokers were defined as those who regularly smoked cigarettes at the time of the survey. BMI was calculated by dividing the participants’ weight (in kilograms) by their height squared (in meters). WC was measured using an inelastic tape at the midpoint of the bottom of the rib cage and the top of the iliac crest. Obesity was defined as a BMI of 25 kg/m^2^ or more [30], and central obesity was defined as a WC greater than 90 cm for men and greater than 80 cm for women.

### Genomic DNA extraction and genotyping

Three *LIPC* SNPs (rs2043085, rs1532085, and rs1800588), that have previously been found consistently associated with various metabolic phenotypes [17, 18, 22, 31, 32], were selected according to the NCBI SNP database (http://www.ncbi.nlm.nih.gov/SNP). Genotyping was then performed using TaqMan SNP Genotyping Assays from Applied Biosystems (ABI; Foster City, CA, USA). For quality control purposes, approximately 10% of the samples were regenotyped in a blinded manner, revealing identical results.

### Laboratory examinations and assays

Laboratory examinations and assays were performed as previously reported [10]. In brief, serum insulin levels were measured using an immunoradiometric assay (Biosource, Nivelles, Belgium). Glucose levels were determined enzymatically using the hexokinase method, and triglyceride levels were measured by automatic enzymatic colorimetry. High-density lipoprotein cholesterol (HDLC) levels were measured enzymatically after phosphotungsten/magnesium precipitation. The HOMA-IR index was calculated as follows: HOMA-IR = fasting insulin (μU/mL) × FPG (mmol/L)/22.5 [33]. According to the previous studies [34, 35], the determination of cut-off values were made on the percentile criterion (66th to 90th according to studies) of values in the general population and the cut-off values ranged from 1.55 to 3.8, with no age or gender differences in HOMA-IR levels under 50 years old. In our study, IR was identified when the HOMA-IR index reached the upper quartile. VAI and LAP were calculated using the following formulas: VAI for men, [WC (cm)/39.68 + (1.88 × BMI)] × (TG/1.03) × (1.31/HDL-C); VAI for women, [WC (cm)/36.58 + (1.89 × BMI)] × (TG/0.81) × (1.52/HDL-C) [4]; LAP for men (WC [cm] − 65) × TG); and LAP for women, (WC [cm] − 58) × TG) [22]. Notably, both the TG and HDL-C levels were expressed in mmol/L. Finally, the TyG-related parameters were calculated as follows: TyG index, Ln [TG (mg/dL) × FPG (mg/dL)/2] [36, 37]; TyG-BMI index, TyG index × BMI; and TyG-WC index, TyG index × WC. Overall, the intra- and inter-assay variability of coefficients was within the range of 1.2–8.2% (Additional file 1: Table S1).

### Statistical analysis

A Chi squared test was used to examine the differences in the categorical variables and to compare allele and genotype frequencies. The clinical characteristics of continuous variables are expressed as mean ± SD and were tested using a two-sample *t* test or analysis of variance. A general linear model was applied to capture the major effect of each polymorphism on clinical phenotype variables, with age, sex, smoking, use of antihypertensive medication, and HDL cholesterol levels with or without serum TG levels as confounding covariates. All biomarker levels were logarithmically transformed prior to statistical analysis to adhere to a normality assumption, and the Bonferroni method was used to correct multiple comparisons where applicable. Values of *P* < 0.05 obtained using two-sided tests were considered statistically significant. Missing data were approached with listwise deletion.

## Results

### Baseline data

The demographic data, clinical biochemical data, lipid profiles, adiposity status, visceral adiposity indicators, and TyG index-related parameters of the participants, stratified by sex, are summarized in Table 1. A significantly higher percentage of men were current smokers (*P* < 0.001) than were women. In addition, men had significantly higher BMIs (*P* < 0.001), WCs (*P* < 0.001), TG (*P* < 0.001), circulating levels of FPG (*P* < 0.001), serum insulin (*P* = 0.01), HOMA-IR (*P* < 0.001), LAP (*P* < 0.001), VAI (*P* < 0.001), TyG index (*P* < 0.001), TyG-BMI index (*P* < 0.001), and TyG-WC index (*P* < 0.001), than did women. By contrast, circulating HDL-C (*P* < 0.001) was lower in men than in women. For the status of glucose metabolism, the proportion of individuals are presented below: fasting glucose levels of 464 subjects are under 100 mg/dL, 108 subjects show fasting glucose levels ranging from 100 to 125 mg/dL and another 20 subjects have greater than 126 mg/dL respectively.

**Table 1:** Clinical and biochemical characteristics of the study population

	Total	Men	Women	*P* value
Number	592	310	282	
Age (years)	45.8 ± 9.7	45.0 ± 9.6	46.7 ± 9.7	0.035
BMI (kg/m^2^)	24.3 ± 3.5	24.9 ± 3.2	23.6 ± 3.7	2.97 × 10^−7^
Waist circumference (cm)	85.0 ± 9.6	87.9 ± 7.8	81.8 ± 10.5	1.05 × 10^−16^
Current smokers (number; %)	115; 19.4	104; 33.5	11; 3.9	4.02 × 10^−21^
Systolic blood pressure^a^ (mmHg)	113.1 ± 16.1	114.0 ± 14.3	112.1 ± 17.8	0.076
Diastolic blood pressure^a^ (mmHg)	75.0 ± 9.9	76.8 ± 9.7	73.1 ± 9.9	6.9 × 10^−6^
HDL-C (mg/dL)	55.4 ± 14.3	49.9 ± 12.0	61.5 ± 14.2	2.16 × 10^−25^
TG (mg/dL)	142.5 ± 119.1	172.9 ± 148.0	109.2 ± 60.1	2.26 × 10^−15^
Fasting plasma glucose (mg/dL)	96.2 ± 22.6	99.0 ± 26.0	93.2 ± 17.8	5.40 × 10^−5^
Fasting serum insulin (µU/mL)	9.2 ± 4.9	9.8 ± 5.6	8.6 ± 3.9	0.005
HOMA-IR	2.2 ± 1.4	2.4 ± 1.6	2.0 ± 1.1	1.09 × 10^−4^
LAP	3560.2 ± 3902.0	4240.1 ± 4780.9	2812.8 ± 2412.0	1.30 × 10^−7^
VAI	196.1 ± 245.0	265.9 ± 308.9	119.4 ± 100.4	8.78 × 10^−29^
TyG index	8.6 ± 0.6	8.8 ± 0.7	8.4 ± 0.5	2.26 × 10^−15^
TyG-BMI	210.3 ± 38.3	220.5 ± 35.4	199.0 ± 38.2	1.24 × 10^−13^
TyG-WC	735.7 ± 116.8	777.3 ± 102.8	690.0 ± 114.3	6.27 × 10^−23^

Continuous variables are presented as mean ± SD. HDL-C, TG, LAP, VAI, TyG, TyG-BMI and TyG-WC values were logarithmically transformed before statistical testing to meet the assumption of normal distributions; however, the untransformed data are shown

*BMI* body mass index, *HDL-C* high-density lipoprotein cholesterol, *TG* triglycerides, *HOMA-IR* homeostasis model assessment of insulin resistance, *LAP* lipid accumulation product, *VAI* visceral adiposity index, *TyG index* the product of triglycerides and fasting glucose, *WC* waist circumference

^a^Only those participants not using antihypertensive drugs were analyzed

### Relationship between LIPC SNPs and adiposity status

To determine the effects of *LIPC* genotypes on adiposity status, we created an additive model by using HDL-C, TG, BMI and WC as variables of interest (Table 2). After adjustment for age, sex, smoking status, and use of antihypertensive medications, all three SNPs were more significantly associated with TG or HDL-C levels after additional adjustment for HDL-C or TG levels (all *P* = 0.012 for HDL-C and *P* = 3.4 × 10^−5^, 2.6 × 10^−5^ and 0.012 for TG, respectively, after the Bonferroni correction).

**Table 2:** Association of LIPC SNPs with adiposity status

SNPs	Genotypes			P*	Adjusted *P*
	MM	Mm	mm		
rs2043085	CC	CT	TT		
BMI (kg/m^2^)	23.7 ± 3.3	24.4 ± 3.6	24.8 ± 3.4	0.002	0.024
WC (cm)	83.6 ± 9.2	85.1 ± 9.7	87.2 ± 9.8	2.8 × 10^−4^	3.4 × 10^−3^
HDL-C (mg/dL)	54.5 ± 14.1	55.9 ± 14.6	55.2 ± 14.2	0.001^#^	0.012
TG (mg/dL)	119.6 ± 65.4	144.8 ± 112.1	169.2 ± 175.7	2.8 × 10^−6^	3.4 × 10^−5^
rs1532085	GG	GA	AA		
BMI (kg/m^2^)	23.8 ± 3.3	24.5 ± 3.6	24.7 ± 3.4	0.006	0.072
WC (cm)	83.6 ± 9.3	85.2 ± 9.6	86.9 ± 9.9	0.001	0.012
HDL-C (mg/dL)	54.5 ± 14.0	55.8 ± 14.5	55.3 ± 14.5	0.001^#^	0.012
TG (mg/dL)	119.6 ± 65.5	144.6 ± 111.5	171.0 ± 179.8	2.2 × 10^−6^	2.6 × 10^−5^
rs1800588	CC	CT	TT		
BMI (kg/m^2^)	24.4 ± 3.5	24.4 ± 3.5	23.6 ± 3.4	0.432	1.0
WC (cm)	85.5 ± 9.8	85.2 ± 9.4	83.4 ± 9.8	0.631	1.0
HDL-C (mg/dL)	53.9 ± 13.1	55.7 ± 14.7	58.3 ± 16.1	0.001^#^	0.012
TG (mg/dL)	135.1 ± 96.1	147.9 ± 139.7	146.8 ± 105.8	0.001	0.012

Abbreviations as in Table 1

*MM* homozygosity of major allele, *Mm* heterozygosity, *mm* homozygosity of minor allele

Adjusted *P*: the P value were shown with Bonferroni corrections, n = 12

P*: adjusted for age, sex, current smoke, hypertension medicine, HDL-C

^#^Adjusted for age, sex, current smoke, hypertension medicine, TG

**Fig. 1:**
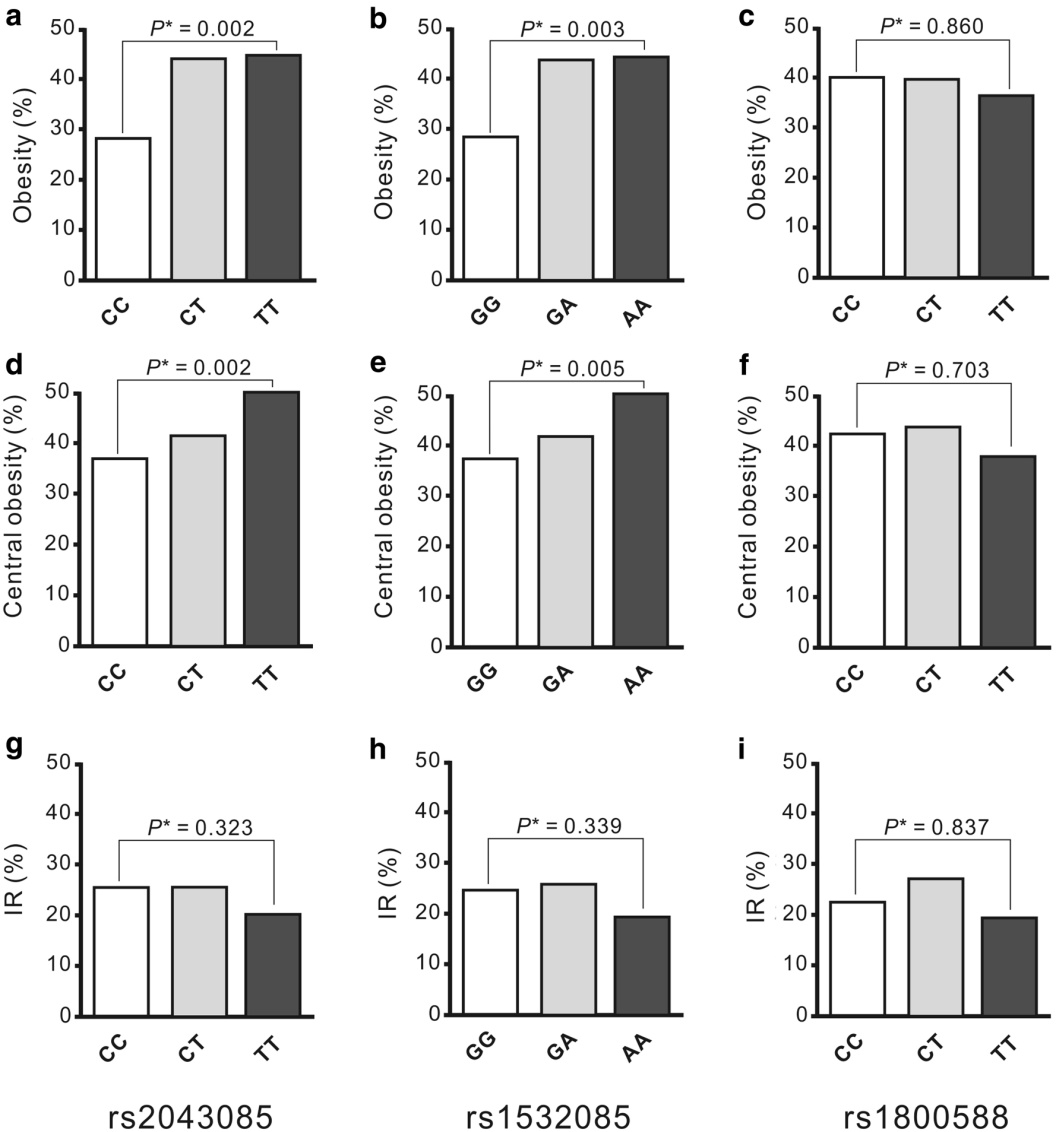
Adiposity status and insulin resistance (IR) according to *LIPC* SNP genotypes. **a**–**f** Significant associations were found between *LIPC* SNPs rs2043085 and rs1532085, but not rs1800588, genotypes and adiposity status, including obesity and central obesity (All *P* < 0.01). **g**–**i** No significant association of *LIPC* genotypes and IR were noted

Moreover, after adjustment for age, sex, smoking status, use of antihypertensive medications, and HDL-C levels, the participants carrying the minor alleles of the studied SNPs exhibited a trend of higher BMIs (*P* = 0.024 and 0.072 for rs2043085 and rs1532085, respectively, after the Bonferroni correction), higher WCs (*P* = 3.4 × 10^−3^, and 0.012 for rs2043085 and rs1532085, respectively, after the Bonferroni correction), and significantly higher frequencies of obesity and central obesity statuses, compared with those carrying the major alleles (Fig. 1). By contrast, no significant association was observed between the rs1800588 genotype and adiposity status.

### Associations between LIPC SNPs and visceral adiposity indicators

To determine whether the *LIPC* genotypes influenced visceral adiposity indicators, we analyzed the LAP and VAI (Table 3). After adjustment for age, sex, smoking status, use of antihypertensive medications, and HDL-C levels with the Bonferroni correction, participants carrying the minor alleles of the rs2043085 and rs1532085 genotypes exhibited a significant trend of higher LAP (*P* = 3.2 × 10^−6^ and 8.1 × 10^−6^ for rs2043085 and rs1532085, respectively) and VAI (*P* = 4.3 × 10^−6^ and 5.2 × 10^−6^ for rs2043085 and rs1532085, respectively). However, the associations became nonsignificant after further adjustment for serum TG levels. By contrast, no significant association between the rs1800588 genotype and visceral adiposity indicators was observed.

**Table 3:** Association of LIPC SNPs with visceral adiposity indicators, TyG index related parameters and insulin resistance

SNPs	Genotypes			P* (adjusted *P*)	P** (adjusted *P*)
	MM	Mm	mm		
rs2043085	CC	CT	TT		
LAP	2863.0 ± 2180.5	3609.6 ± 3537.8	4451.0 ± 5943.0	1.8 × 10^−7^ (3.2 × 10^−6^)	0.016 (0.288)
VAI	183.8 ± 132.6	219.0 ± 203.6	266.4 ± 381.5	2.4 × 10^−7^ (4.3 × 10^−6^)	0.011 (0.198)
TyG	8.5 ± 0.6	8.6 ± 0.7	8.8 ± 0.7	1.7 × 10^−5^ (3.1 × 10^−4^)	0.542
TyG-BMI	202.5 ± 35.0	211.7 ± 39.7	217.9 ± 38.1	2.0 × 10^−5^ (3.6 × 10^−4^)	0.026 (0.468)
TyG-WC	713.2 ± 109.5	736.9 ± 118.3	765.7 ± 117.6	1.4 × 10^−6^ (2.5 × 10^−5^)	0.013 (0.234)
HOMA-IR	2.2 ± 1.3	2.2 ± 1.4	2.3 ± 1.5	0.422	0.818
rs1532085	GG	GA	AA		
LAP	2876.5 ± 2181.0	3613.7 ± 3519.0	4470.7 ± 6085.4	4.5 × 10^−7^ (8.1 × 10^−6^)	0.040 (0.720)
VAI	184.1 ± 132.1	218.5 ± 202.5	270.1 ± 390.5	2.9 × 10^−7^ (5.2 × 10^−6^)	0.029 (0.522)
TyG	8.5 ± 0.6	8.6 ± 0.7	8.8 ± 0.7	1.0 × 10^−5^ (1.8 × 10^−4^)	0.701
TyG-BMI	202.8 ± 35.1	211.9 ± 39.5	217.3 ± 38.8	5.1 × 10^−5^ (9.2 × 10^−4^)	0.051 (0.918)
TyG-WC	713.4 ± 110.4	738.4 ± 117.3	763.2 ± 119.7	4.7 × 10^−6^ (8.5 × 10^−5^)	0.034 (0.612)
HOMA-IR	2.2 ± 1.3	2.2 ± 1.4	2.3 ± 1.5	0.449	0.766
rs1800588	CC	CT	TT		
LAP	3385.6 ± 3198.8	3746.4 ± 4593.1	3514.7 ± 3246.5	0.026 (0.468)	0.288
VAI	208.8 ± 184.7	230.6 ± 290.1	207.7 ± 176.1	0.003 (0.054)	0.163
TyG	8.6 ± 0.6	8.6 ± 0.7	8.8 ± 0.7	4.6 × 10^−4^ (0.008)	0.521
TyG-BMI	210.2 ± 37.4	211.9 ± 39.0	205.6 ± 39.2	0.455	0.242
TyG-WC	735.2 ± 116.2	739.7 ± 115.5	726.4 ± 125.0	0.182	0.303
HOMA-IR	2.2 ± 1.4	2.2 ± 1.4	2.2 ± 1.5	0.219	0.632

Abbreviations as in Table 1

*MM* homozygosity of major allele, *Mm* heterozygosity, *mm* homozygosity of minor allele

Adjusted *P*: the P value were shown with Bonferroni corrections, n = 18

P*: adjusted for age, sex, current smoke, hypertension medicine, HDL-C; P**: Further adjusted for TG

### Associations between LIPC SNPs and TyG-related parameters and IR status

To determine whether the *LIPC* genotypes influenced TyG-related parameters, we analyzed the TyG, TyG-BMI, and TyG-WC indices (Table 3). After adjustment for age, sex, smoking status, use of antihypertensive medications, and HDL-C levels with the Bonferroni correction, the participants carrying the minor alleles of the rs2043085 and rs1532085 genotypes tended to have a significantly higher TyG index (*P* = 3.1 × 10^−4^ and 1.8 × 10^−4^ for rs2043085 and rs1532085, respectively), TyG-BMI index (*P* = 3.6 × 10^−4^ and 9.2 × 10^−4^ for rs2043085 and rs1532085, respectively), and TyG-WC index (*P* = 2.5 × 10^−5^ and 8.5 × 10^−5^ for rs2043085 and rs1532085, respectively). In addition, significant associations were observed between the rs1800588 genotype and TyG index (*P* = 0.008 after the Bonferroni correction); however, these associations became nonsignificant after further adjustment for serum TG levels. By contrast, no significant association between the rs1800588 genotype and the TyG-BMI and TyG-WC indices was observed. Furthermore, no significant association between *LIPC* SNPs and the HOMA-IR and IR statuses was noted (Table 3 and Fig. 1).

### Associations between LIPC SNPs and each quartile of the visceral adiposity indicators and TyG index-related parameters

To confirm the effect of *LIPC* genotypes on the visceral adiposity indicators and TyG index-related parameters, we further analyzed the frequencies of the additive model of *LIPC* genotypes in each quartile of the studied parameters (Additional file 1: Table S2). After adjustment for age, sex, smoking status, use of antihypertensive medications and HDL-C levels with the Bonferroni correction, significant *P* values were obtained for the rs2043085 and rs1532085 SNPs (all *P* < 0.01). The participants carrying the minor alleles exhibited a trend of higher frequencies in the upper quartiles of the studied parameters. By contrast, no significant difference in the rs1800588 genotype frequencies was observed between the quartiles of the studied parameters.

## Discussion

This study investigated the association between *LIPC* SNPs and adiposity status, visceral adiposity indicators, and TyG index-related parameters, and HOMA-IR in the Taiwanese population. Our data revealed at least a trend of significant association between two *LIPC* SNPs, rs2043085 and rs1532085, and BMI, WC, and adiposity status. In addition, these *LIPC* SNPs were associated with visceral adiposity indicators and TyG index-related parameters, either in continuous variables or in quartiles which were mediated by serum TG levels. The pleiotropic associations further support a complex interaction between *LIPC* SNPs and the risk of metabolic syndromes, diabetes mellitus and future atherosclerotic cardiovascular disease.

Previous studies have reported controversial results on the association between HL and obesity. For example, mice lacking HL were reported to be lean and protected against diet-induced obesity and hepatic steatosis in one study [38]. The influence of *LIPC* alleles on obesity was investigated through a reciprocal hemizygosity analysis [39]. Additionally, several studies have revealed that *LIPC* SNPs, mostly promoter SNPs, are not associated with BMI. However, one previous study [29] demonstrated the association between *LIPC* promoter SNPs and BMI. Mägi et al. [40] reported that the rs2043085 SNP was the most significant SNP in the *LIPC* locus associated with lipid traits and BMI through a reverse regression approach by using software for correlated phenotype analysis (SCOPA) and META-SCOPA software. Our data revealed that SNPs rs2043085 and rs1532085 were significantly associated with adiposity status. Differential associations between the studied SNPs in adiposity status are interesting. SNP rs1800588 is the promoter *LIPC* SNP that has been most widely reported to be associated with various phenotypes [17–21]. *LIPC* is expressed only in the liver, and both rs1532085 and rs1800588 were associated with the expression of *LIPC* [41]. *LIPC* SNP rs2043085 exhibited high linkage disequilibrium with rs1532085 and either of them has been shown to be the most significant SNP in the *LIPC* locus associated with various phenotypes in several genome-wide association studies (GWASs) [26, 42, 43]. Using a bivariate genome-wide approach for seven studies of the STAMPEED consortium, comprising 22,161 participants of European ancestry, Kraja et al. [26] also showed variants in the *LIPC* gene as one of the loci associated with metabolic syndrome. Our data suggested that the haplotype block, combining all the three studied SNPs, may be more crucial for future studies as a marker for atherosclerotic cardiovascular disease.

The combined associations of *LIPC* SNPs with insulin surrogate markers that based on both metabolic and adiposity status are noteworthy. Shared common variants have similarly been reported between lipid genes and glucose metabolism, inflammation, BMI, cardiometabolic traits, and coronary artery disease [20, 40, 44–46]. Pickrell et al. [21] developed a method of detecting pairs of traits that exhibited an asymmetry in the effect sizes of associated variants, which is more consistent with a causal relationship between the traits and the authors indicated that an elevated BMI causally increases TG levels, but the reverse is not true. By contrast, we have previously demonstrated that *LIPC* SNPs were associated with serum TG and HDL cholesterol levels independently of the BMI [17]. These results suggest the independence of association between *LIPC* SNPs and metabolic and adiposity status.

The genetic determinants of the visceral adiposity indicators and TyG index-related parameters have not been previously reported. These parameters are complex traits derived from simple phenotypes, including metabolic phenotypes with or without adiposity status, with the serum TG level as the most common component of all indices. Thus, a polymorphism associated with both adiposity status and serum TG levels may be associated with these parameters. Furthermore, the associations became nonsignificant after adjustment for serum TG levels. Because of the involvement of serum TG levels in each parameter, other genetic determinants of serum TG levels may also be the genetic determinants of these parameters; however, further research is required to confirm this phenomenon.

The term pleiotropy is used to describe the phenomenon by which genetic variation at a single locus exerts an effect on more than one phenotype. Pleiotropy observed in genetic association studies can provide insight into the shared biology underlying a spectrum of phenotypes. Pleiotropy may be caused by the third variable with mediational, interactive, reverse causal, or suppressive effect [47–49]. By using the National Human Genome Research Institute’s catalog of a published GWAS, Sivakumaran et al. [50] reported that 4.6% of the SNPs and 16.9% of the genes exhibited the cross-phenotype effect. However, with the growing number of GWASs, this effect is likely to be underestimated. Some chromosomal regions, including the gene loci near *LIPC*, appear to be particular foci for association with multiple phenotypes [20, 21]. Moreover, different *LIPC* SNPs have been associated with levels of circulating malondialdehyde-modified low-density lipoprotein [22]; phospholipids and sphingolipids [51]; folate and vitamin E [52]; BMI [29, 39]; metabolic syndrome [26]; and diseases such as advanced age-related macular degeneration [43, 53, 54], coronary artery disease [18], and myocardial infarction [19]. Our data further revealed that the *LIPC* SNPs are associated with adiposity status, visceral adiposity indicators, and TyG index-related parameters in the Taiwanese population. Visceral obesity has been shown to be associated with IR and a high risk of developing type 2 diabetes after myocardial infarction [55]. Several human studies also indicate that these surrogated markers for IR may identify individuals at a high risk of developing cardiovascular events and mortality [56–62]. Thus, our findings implicated that the genetic determinants of these parameters is crucial in providing a method for identifying high-risk populations of cardiovascular diseases for preventive medicine.

By contrast, IR is influenced by multiple factors, including lipid profiles, obesity and inflammation, in which *LIPC* SNPs are not associated with circulating inflammatory markers in our study population (data not shown). We also found no significant association between any the studied *LIPC* SNPs and IR in term of HOMA-IR.

The main limitation of this study is its medium sample size and relatively low number of genotyped participants. This limitation may be more marked because a complex trait was central to this analysis. Nevertheless, such associations may be attributed to the mediational effect of serum TG levels. Furthermore, only three *LIPC* SNPs were analyzed, which suggests an incomplete coverage of *LIPC* SNPs; hence, the study did not represent all genetic variations of *LIPC*.

## Conclusions

This study provided the first evidence of genetic determinants of various visceral adiposity indicators and TyG index-related parameters. Previous studies have demonstrated that the genetic determinants of serum TG levels determine the risk of coronary artery disease [21, 63]. Additional studies may be necessary to elucidate whether the genetic determinants of serum TG levels are also crucial in pleiotropic genetic associations for visceral adiposity indicators and TyG index-related parameters through mediational effects.

## Additional file

**Additional file 1: Table S1.** Inter- and intra-assay variability measures of the biochemistry. **Table S2.** Associations of the LIPC SNPs with each quartile of visceral adiposity indicators and TyG index related parameters.

## Abbreviations

SNPs: single nucleotide polymorphisms
BMI: body mass index
WC: waist circumference
IR: insulin resistance
HDL-C: high-density lipoprotein cholesterol
LDL-C: low-density lipoprotein cholesterol
TG: triglycerides
LAP: lipid accumulation product
VAI: visceral adiposity index
TyG index: the product of TG and fasting plasma glucose levels
TyG-BMI: TyG index with body mass index
TyG-WC: TyG index with waist circumference
