# Supplementary material for: LIPC variants as genetic determinants of adiposity status, visceral adiposity indicators, and triglyceride-glucose (TyG) index-related parameters mediated by serum triglyceride levels
Source: Diabetol Metab Syndr. 2018 Nov 6;10:79. doi: 10.1186/s13098-018-0383-9 (PMC6218991; doi:10.1186/s13098-018-0383-9)
Supplement: Supplementary file 1 — Additional file 1: Table S1. Inter- and intra-assay variability measures of the biochemistry. Table S2. Associations of the LIPC SNPs with each quartile of visceral adiposity indicators and TyG index related parameters. [file 13098_2018_383_MOESM1_ESM.doc]

**Table S1.** *Inter- and intra-assay variability measures of the biochemistry*

| Biochemistry | Source | Intra-assay | Inter-assay |
| --- | --- | --- | --- |
| HDL-cholesterol (mg/dL) | Serum | CV = 4.0% | CV = 4.1% |
| Triglyceride (mg/dL ) | Serum | CV = 2.1% | CV = 3.4% |
| Fasting plasma glucose (mg/dl) | Plasma | CV = 1.2% | CV = 1.4% |
| Fasting serum insulin (μU/ml) | serum | CV = 8.1% | CV = 8.2% |

CV: coefficient of variation

**Table S2.** *Associations of the LIPC SNPs with each quartile of visceral adiposity indicators and TyG index related parameters*

|  | | rs2043085 | | |  | rs1532085 | | |  | rs1800588 | | |  |
| --- | --- | --- | --- | --- | --- | --- | --- | --- | --- | --- | --- | --- | --- |
|  |  | CC | CT | TT | *P^*^*  (adjusted *P*) | GG | GA | AA | *P^*^*  (adjusted *P*) | CC | CT | TT | *P^*^*  (adjusted *P*) |
| VAI | Q1,≦89 | 49 (29.7) | 78 (26.6) | 20 (16.1) | 0.0001 | 50 (29.6) | 77 (26.0) | 20 (16.9) | 0.0002 | 57 (24.8) | 67 (24.3) | 23 (29.9) | 0.005 |
|  | Q2, 89-151 | 33 (20.0) | 76 (25.9) | 33 (26.6) | (0.0018) | 34 (20.1) | 78 (26.0) | 31 (26.3) | (0.0036) | 65 (28.3) | 63 (22.8) | 15 (19.5) | (0.09) |
|  | Q3,152-270 | 47 (28.5) | 64 (21.8) | 35 (28.2) |  | 48 (28.4) | 66 (22.3) | 32 (27.1) |  | 52 (22.6) | 75 (27.2) | 19 (24.7) |  |
|  | Q4,≧271 | 36 (21.8) | 75 (25.6) | 36 (29.0) |  | 37 (21.9) | 75 (25.3) | 35 (29.7) |  | 56 (24.3 | 71 (25.7) | 20 (26.0) |  |
| LAP | Q1,≦1475 | 52 (31.5) | 70 (23.9) | 22 (17.7) | 4.5 × 10^-6^ | 53 (31.4) | 70 (23.6) | 22 (18.6) | 2.6 × 10^-5^ | 62 (27.0) | 61 (22.1) | 22 (28.6) | 0.042 |
|  | Q2,1476-2732 | 39 (23.6) | 82 (28.0) | 24 (19.4) | (8.1 × 10^-5^) | 40 (23.7) | 81 (27.4) | 24 (20.3) | (4.7 × 10^-4^) | 56 (24.3) | 70 (25.4) | 19 (24.7) | (0.756) |
|  | Q3,2733-4232 | 41 (24.8) | 70 (23.9) | 37 (29.8) |  | 41 (24.3) | 74 (25.0) | 33 (28.0) |  | 57 (24.8) | 75 (27.2) | 16 (20.8) |  |
|  | Q4,≧4233 | 33 (20.0) | 71 (24.2) | 41 (33.1) |  | 35 (20.7) | 71 (24.0) | 39 (33.1) |  | 55 (23.9) | 70 (25.4) | 20 (26.0) |  |
| TyG-index | Q1,≦8.0 | 50 (30.3) | 75 (25.6) | 19 (15.3) | 0.0003 | 51 (30.2) | 75 (25.3) | 19 (16.1) | 0.0003 | 62 (27.0) | 65 (23.6) | 18 (23.4) | 0.001 |
|  | Q2,8.1-8.4 | 38 (23.0) | 75 (25.6) | 33 (26.6) | (0.0054) | 40 (23.7) | 76 (25.7) | 30 (25.4) | (0.0054) | 64 (27.8) | 63 (22.8) | 19 (24.7) | (0.018) |
|  | Q3,8.5-8.8 | 41 (24.8) | 72 (24.6) | 31 (25.0) |  | 41 (24.3) | 74 (25.0) | 29 (24.6) |  | 49 (21.3) | 76 (27.5) | 19 (24.7) |  |
|  | Q4,≧8.9 | 36 (21.8) | 71 (24.2) | 41 (33.1) |  | 37 (21.9) | 71 (24.0) | 40 (33.9) |  | 55 (23.9) | 72 (26.1) | 21 (27.3) |  |
| TyG-BMI | Q1,≦182.2 | 50 (30.3) | 74 (25.3) | 21 (16.9) | 9.4 × 10^-5^ | 51 (30.2) | 73 (24.7) | 21 (17.8) | 0.0002 | 52 (22.6) | 67 (24.3) | 26 (33.8) | 0.610 |
|  | Q2,182.3-208 | 50 (30.3) | 66 (22.5) | 28 (22.6) | (1.7 × 10^-4^) | 51 (30.2) | 67 (22.6) | 27 (22.9) | (0.0036) | 66 (28.7) | 62 (22.5) | 17 (22.1) |  |
|  | Q3,208.1-234 | 30 (18.2) | 76 (25.9) | 41 (33.1) |  | 31 (18.3) | 78 (26.4) | 38 (32.2) |  | 55 (23.9) | 76 (27.5) | 16 (20.8) |  |
|  | Q4≧,1234.5 | 35 (21.2) | 77 (26.3) | 34 (27.4) |  | 36 (21.3) | 78 (26.4) | 32 (27.1) |  | 57 (24.8) | 71 (25.7) | 18 (23.4) |  |
| TyG-WC | Q1,≦644 | 51 (30.9) | 74 (25.3) | 18 (14.5) | 1.2 × 10^-5^ | 52 (30.8) | 74 (25.0) | 18 (15.3) | 6.5 × 10^-5^ | 60 (26.1) | 61 (22.1) | 23 (29.9) | 0.207 |
|  | Q2,645-733 | 45 (27.3) | 75 (25.6) | 26 (21.0) | (2.2 × 10^-4^) | 46 (27.2) | 74 (25.0) | 26 (22.0) | (1.2 × 10^-3^) | 52 (22.6) | 73 (26.4) | 21 (27.3) |  |
|  | Q3,734-811 | 33 (20.0) | 70 (23.9) | 44 (35.5) |  | 34 (20.1) | 72 (24.3) | 41 (34.7) |  | 63 (27.4) | 71 (25.7) | 13 (16.9) |  |
|  | Q4,≧812 | 36 (21.8) | 74 (25.3) | 36 (29.0) |  | 37 (21.9) | 76 (25.7) | 33 (28.0) |  | 55 (23.9) | 71 (25.7) | 20 (26.0) |  |

Abbreviations as in Table 1

Q: Quartile; *P^*^*: adjusted for age, sex, current smoke, hypertension medicine, HDL-C;

Adjusted *P*: the P value were shown with Bonferroni corrections, n = 18; Data are expressed as numbers (percentages)
